# Extracellular glutamate is not modulated by cannabinoid receptor activity

**DOI:** 10.1038/s41598-024-75962-5

**Published:** 2024-11-06

**Authors:** Delia N. Chiu, Brett C. Carter

**Affiliations:** ENI-G, a Joint Initiative of the University Medical Center Göttingen and the Max Planck Institute for Multidisciplinary Sciences, 37077 Göttingen, Germany

**Keywords:** Neuroscience, Cellular neuroscience, Neuronal physiology, Synaptic transmission

## Abstract

Cannabinoid receptor activation has been proposed to trigger glutamate release from astrocytes located in cortical layer 2/3. Here, we measure the basal concentration of extracellular glutamate in layer 2/3 of mouse somatosensory cortex and find it to be 20–30 nM. We further examine the effect of cannabinoid receptor signaling on extracellular glutamate, and find no evidence for increased extracellular glutamate upon cannabinoid receptor agonist application.

## Main text

At excitatory synapses between layer 4 (L4) spiny stellate neurons and layer 2/3 (L2/3) pyramidal neurons in rodent primary somatosensory cortex (barrel cortex), cannabinoid receptor signaling is required for the induction of spike-timing dependent long-term depression (tLTD) and the development of receptive fields^1–3^.

In the central nervous system, the effects of endogenous and exogenous cannabinoids are mediated by Type 1 cannabinoid receptors (CB1Rs), the most abundant G-protein coupled receptors in the brain^4^. Canonical CB1R signaling suppresses synaptic release via retrograde transmission of endocannabinoids^5^. CB1R signaling is also involved in LTD induction in diverse brain areas including hippocampus, cortex, striatum, and cerebellum^6^. CB1Rs can thus reduce the likelihood of neurotransmitter release in both the short and long term, and these effects are usually mediated by presynaptic CB1Rs.

There are conflicting data regarding whether CB1Rs are expressed presynaptically at L4-L2/3 synapses^7,8^. However, astrocytes also express CB1Rs, including in L2/3^9,10^. Unlike neuronal CB1Rs, astrocyte CB1Rs tend to be G_q_-coupled, such that their activation is linked to increases in intracellular calcium through IP_3_R signaling^11^. In hippocampus^9^ and layer 2/3 of somatosensory cortex^3,12^, CB1R activation elicits calcium transients in astrocytes during exogenous agonist application or during patterns of activity that induce tLTD^3^. In L2/3, these calcium signals are thought to trigger astrocytic vesicular release of glutamate, which in turn is sensed by neuronal NMDARs. A more recent study^10^ found that CB1R activation increased extracellular glutamate both in cortical astrocyte cultures and also in vivo in medial prefrontal cortex, albeit on a much longer time scale.

If CB1R activation increases extracellular glutamate, this would have consequences for synapse and circuit function. Excessive extracellular glutamate impairs rapid neuronal signaling by desensitizing receptors^13^, and chronic glutamate exposure is considered the primary driver of neuronal excitotoxicity^14^, and has been hypothesized to contribute to a large number of pathologies^15^. Crucially, L2/3 pyramidal neurons in barrel cortex display NMDAR-mediated current at membrane potentials generally regarded as non-permissive in the presence of extracellular Mg^2+^ (V_m_ = -70 to -80 mV)^16,17^. Given the sensitivity of L2/3 pyramidal neurons to glutamate, as well as the importance of preserving the ability to discriminate signal from noise in a sensory circuit, cannabinoid receptor-induced glutamate release would have important and heretofore unexplored consequences for synaptic transmission. We therefore sought to investigate extracellular glutamate in L2/3 of barrel cortex and its modulation by cannabinoid receptor activation.

**Fig. 1:**
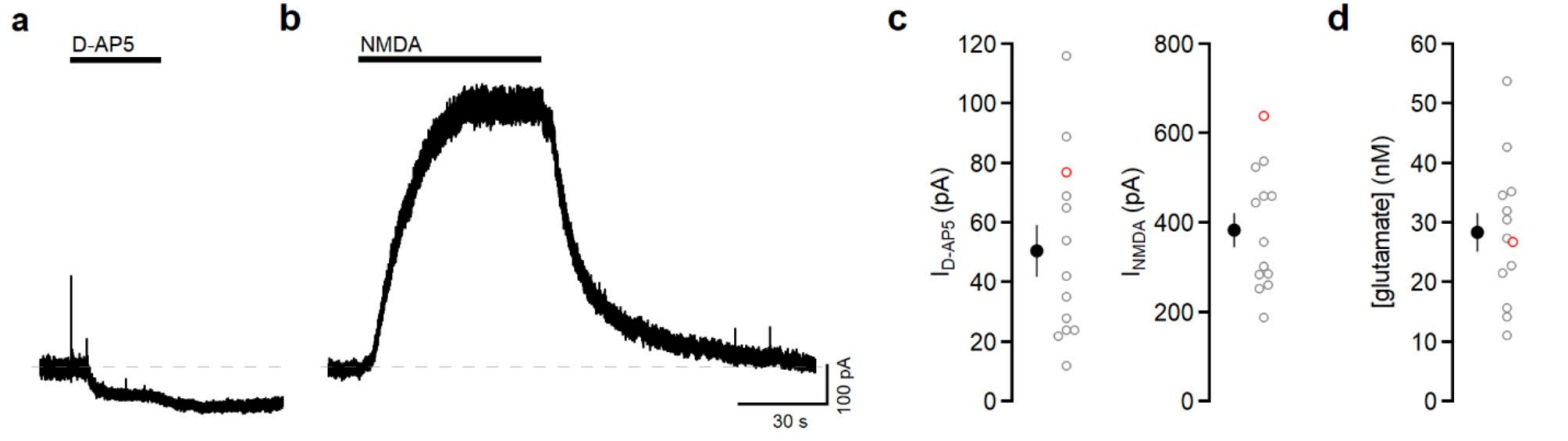
L2/3 pyramidal neuron NMDARs report nanomolar extracellular glutamate. (**a**) Voltage-clamp recording from a L2/3 pyramidal neuron at +30 mV. D-AP5 (50 µM) reduced the outward holding current. Dashed line denotes baseline. (**b**) In the same cell, subsequent application of NMDA (5 µM) elicited an outward current. (**c**) Summary of the currents blocked by D-AP5 and evoked by NMDA (*n* = 13 cells from 10 mice). (**d**) For each cell, the glutamate concentration was derived by normalizing the current blocked by DAP5 to the current generated by NMDA (28.3 ± 0.3 nM, *n* = 13). The red open symbols correspond to the example traces shown in (**a)** and (**b**). In these and all subsequent summary graphs, open symbols denote individual data points and closed symbols represent mean ± s.e.m.

To determine the basal extracellular glutamate concentration in L2/3 of mouse barrel cortex, we made whole-cell recordings from L2/3 pyramidal neurons in acute brain slices. Neurons were voltage-clamped at + 30 mV in physiological ACSF to relieve Mg^2+^ block of NMDAR channels only in the recorded cell. NMDAR currents were isolated by including in the superfusate TTX, NBQX, and picrotoxin. D-serine (10 µM) was present to ensure NMDAR glycine binding sites were saturated. In these conditions, application of the competitive NMDAR antagonist D-AP5 (50 µM; Fig. 1a) blocks a component of the standing current due to the activation of NMDARs by ambient extracellular glutamate. On average, D-AP5 reduced the standing current by 50.5 ± 8.6 pA (*n* = 13 cells from 10 mice, Fig. 1c).

The size of the NMDAR current depends on both the concentration of glutamate and the number of receptors present. Application of a known concentration of glutamate would provide information about the total number of NMDARs. However, transporters present in the slice prevent exogenously applied glutamate from reaching NMDARs^18,19^. To overcome this problem, in each recording we measured the current evoked by 5 µM NMDA (Fig. 1b), a non-transported NMDAR agonist. Taking into account the different potency and efficacy of the two NMDAR agonists^19,20^ allowed us to convert the D-AP5-sensitive current into a fraction of the maximal glutamate current. We used a previously derived glutamate dose-response curve^19^ to determine that this corresponds to 28.3 ± 3.3 nM glutamate (*n* = 13, Fig. 1). This is in close agreement with the concentration reported by NMDARs in hippocampus, nucleus accumbens, and medial nucleus of trapezoid body^19,21–23^.

**Fig. 2:**
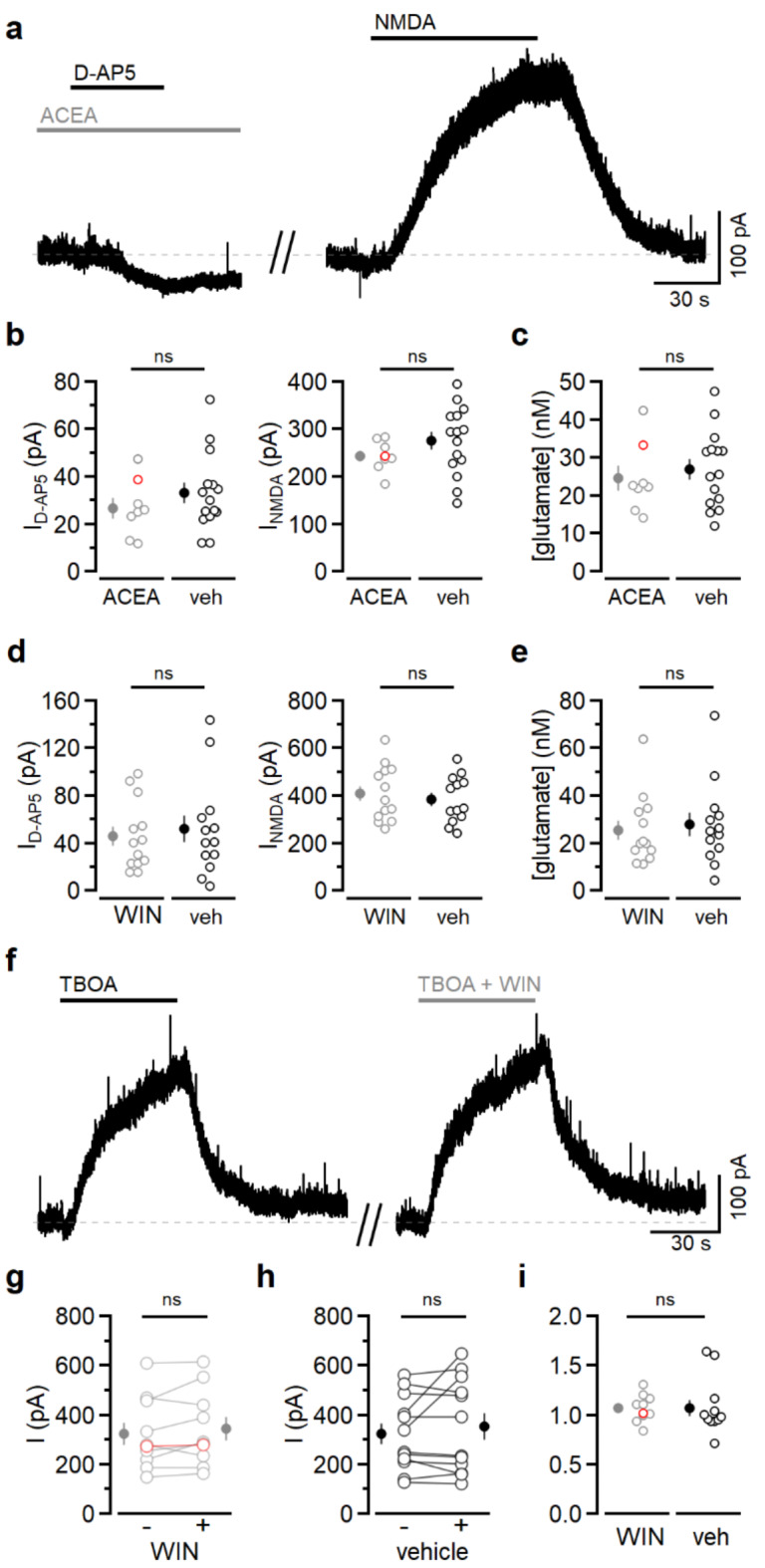
CB1R activation does not alter extracellular glutamate. (**a**) Voltage-clamp recording from a L2/3 pyramidal neuron at +30 mV. Left, ACEA (1 µM) was applied for 2.5 min prior to D-AP5 (50 µM). Right, after washout of ACEA and D-AP5, application of NMDA (5 µM) elicited an outward current. Dashed line denotes baseline. (**b**) Left, the current blocked by D-AP5 when ACEA (grey symbols, *n* = 8 cells from 2 mice) or vehicle (black symbols, *n* = 15 cells from 3 mice) was applied for 2.5–3 min prior to D-AP5 (*P* = 0.34). Right, the NMDA-induced currents were also similar (*P* = 0.25). The red open symbols correspond to the example traces shown in (**a**). (**c**) The extracellular glutamate concentration was the same in ACEA and vehicle (24.5 ± 3.2 nM versus 27.0 ± 2.6 nM, *P* = 0.57). (**d**) As in (**b**), with 5 µM WIN (grey symbols, *n* = 13 cells from 3 mice) versus vehicle (black symbols, *n* = 13 cells from 5 mice; D-AP5-sensitive current: *P* = 0.34; NMDA current: *P* = 0.34). (**e**) The extracellular glutamate concentration was the same in WIN and vehicle (25.5 ± 4.1 nM versus 27.9 ± 4.9 nM, *P* = 0.70). (**f**) Voltage-clamp recording from a L2/3 pyramidal neuron at + 30 mV. TBOA (100 µM) led to an outward current. After washout, TBOA and WIN (5 µM) were applied together. (**g**) Current in TBOA alone or in TBOA + WIN (*n* = 10 cells from 5 mice, *P* = 0.18). The red open symbols correspond to the example shown in (**f**). (**h**) Current in TBOA alone or in TBOA + vehicle (*n* = 12 cells from 6 mice, *P* = 0.31). (**i**) Currents obtained in the presence of WIN or vehicle normalized to the currents measured in TBOA only (*P* = 0.98). The red open symbols in (**g**) and (**i**) correspond to the example shown in (**f**). The experiments summarized in g and h were analyzed using paired *t*-tests. All other comparisons were done with unpaired *t*-tests.

We next tested whether the synthetic CB1R agonists ACEA (Fig. 2a-c) or WIN (Fig. 2d,e) altered extracellular glutamate concentration compared to vehicle (ethanol or DMSO, respectively), with experimenter blind to condition. In a previous study^3^, the effect of CB1R activation on astrocytic calcium transients required over two minutes of agonist presentation, so to maximize the likelihood of detecting an effect of CB1R activation on glutamate, solution containing ACEA, WIN, or vehicle was applied for at least 2.5 min before blocking NMDAR current with D-AP5. ACEA (1 µM) had no effect on D-AP5-sensitive current or NMDA-evoked current (Fig. 2b). Accordingly, the extracellular glutamate concentration was the same in ACEA and vehicle (Fig. 2c). Similar results were obtained using WIN (5 µM; Fig. 2d,e). Because depolarization can trigger endocannabinoid synthesis and release^24^, the lack of effect of CB1R agonists could reflect occlusion due to endocannabinoid activation of the receptors. In our experiments, postsynaptic calcium entry is limited by both inactivation (due to sustained depolarization of the neuron) and the presence of voltage-dependent calcium channel antagonists. To test whether CB1R-dependent glutamate release contributed to our basal extracellular glutamate estimates, we measured D-AP5 sensitive current in the presence of the CB1R antagonist AM251 (1 µM). AM251 was applied for at least three minutes before cells were clamped at + 30 mV. In these experiments, the extracellular glutamate concentration was 21.2 ± 2.6 nM (*n* = 8 cells from 3 mice; versus 28.3 ± 3.3 nM, *P* = 0.15, *t*-test) which indicates that CB1R activation does not contribute to extracellular glutamate measurements using this technique. Although there was no detectable increase in extracellular glutamate in the continued presence of CB1R agonists, one caveat is that glutamate uptake was intact during these experiments.

Given the proposed extrasynaptic origin of CB1R-mediated glutamate release^3^, transporters could buffer glutamate before NMDARs detect it. To address this possibility, we used the non-substrate glutamate transporter blocker DL-TBOA (TBOA) to inhibit glutamate uptake^25^.

TBOA (100 µM) led to a reversible increase in standing current (Fig. 2h), reflecting the activation of NMDARs by accumulation of extracellular glutamate^21,22^. To test whether CB1R activation further increases extracellular glutamate, we applied a second solution containing 100 µM TBOA and either WIN (5 µM) or vehicle, with experimenter blind to condition (Fig. 2h). Neither WIN (Fig. 2g) nor vehicle (Fig. 2h) changed the size of the current evoked by TBOA. To compare the two conditions, currents were normalized to the current measured in TBOA alone. The current evoked by TBOA in the presence of WIN was indistinguishable from the current evoked by TBOA and vehicle (Fig. 2i). Although the glutamate concentration was not directly measured in these experiments, the current measured in TBOA is unlikely to represent maximal activation of NMDARs. First, the current was roughly the same magnitude as the current elicited by 5 µM NMDA in other sets of experiments; this dose is well below the EC_50_ (37.7 µM) for NMDA^19^. Second, in a subset of recordings, 1 µM glutamate was applied with TBOA at the end of the experiment, which increased NMDAR-mediated current relative to TBOA alone (WIN group: 461.4 ± 56.6 pA versus 328.7 ± 51.1 pA, *n* = 9 cells from 5 mice, *P* = 0.0003, paired *t*-test; vehicle group: 458.4 ± 58.8 pA versus 318.9 ± 48.2 pA, *n* = 10 cells from 6 mice, *P* = 0.009, paired *t*-test), indicating that NMDARs were not saturated. These experiments indicate that application of cannabinoid receptor agonist also does not lead to measurable increases in extracellular glutamate concentration within seconds, and that glutamate uptake did not mask the effect of CB1R activation.

**Fig. 3:**
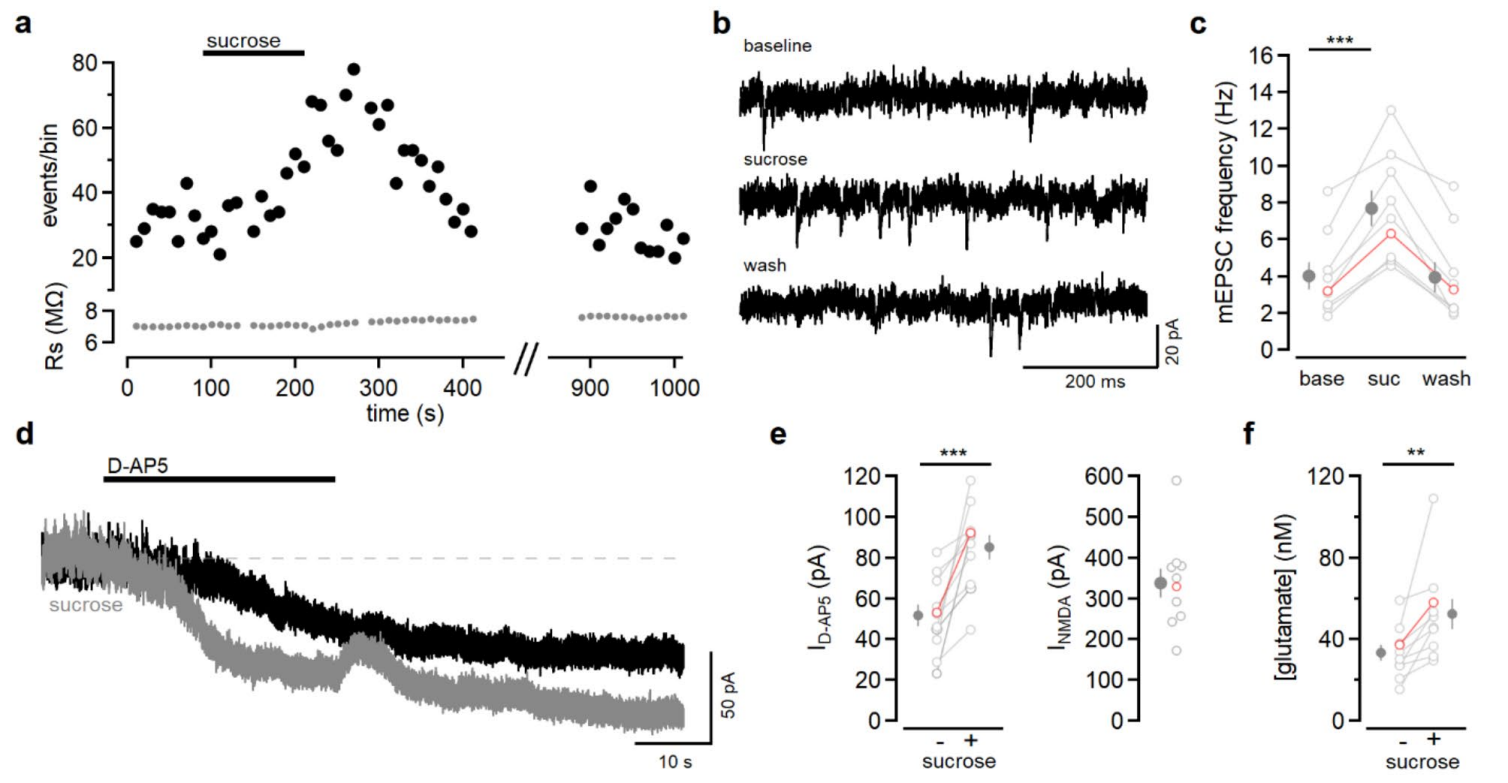
Sucrose increases mEPSC frequency and extracellular glutamate. (**a**) Summary of an experiment monitoring mEPSCs before, during, and after bath application of ACSF containing 40 mM sucrose. Each point on the graph represents the measurement from one recording block of 10 s. Black symbols represent the number of mEPSCs and grey symbols represent the series resistance. (**b**) Example traces from experiment shown in (**a**). (**c**) Summary of mEPSC frequency before (base), during (suc), and 8.5 min after (wash) sucrose solution application (*n* = 9 cells from 5 mice). Frequency increased in sucrose (*P* = 0.0001). The red open symbols correspond to the example shown in (**a**) and (**b**). (**d**) Voltage-clamp recording from a L2/3 pyramidal neuron at +30 mV. D-AP5 (50 µM) reduces the outward standing current in control (black trace) and in sucrose-containing solution (grey). (**e**) The current blocked by D-AP5 was greater when sucrose was present (*n* = 11 cells from 6 mice, *P* = 0.001). The response to 5 µM NMDA was measured in 10 of 11 experiments. (**f**) The level of NMDAR activation corresponded to a basal glutamate concentration of 33.4 ± 3.9 nM and increased to 52.4 ± 7.2 nM in sucrose (*n* = 10 cells from 6 mice, *P* = 0.009). The red open symbols correspond to the example traces shown in (**d**). Statistical analysis was done with paired *t*-tests.

To test whether this experimental approach is sensitive enough to detect a change in extracellular glutamate under conditions of increased vesicular release, we took advantage of the fact that hyperosmotic extracellular solution promotes vesicle release^26,27^. Supplementing ACSF with 40 mM sucrose reversibly increased the frequency of miniature excitatory postsynaptic currents (mEPSCs) measured in L2/3 neurons (Fig. 3a, b). Sucrose increased mEPSC frequency (Fig. 3b, c; 7.7 ± 1.0 Hz versus 4.0 ± 0.7 Hz, *n* = 9 cells from 5 mice, *P* = 0.0001, paired *t*-test) and had no effect on mEPSC amplitude (sucrose: -12.9 ± 0.8 pA, baseline: -12.9 ± 0.8 pA, *n* = 9, *P* = 0.78, paired *t*-test).

To determine whether this modest increase in absolute mEPSC frequency was detectible using the standing current approach, we measured the D-AP5-sensitive current (Fig. 3d) before and after sucrose application (Fig. 3d). Finally, we measured the NMDAR-evoked current to normalize the D-AP5-sensitive currents and estimate the glutamate concentration. Although glutamate uptake was intact, the current blocked by D-AP5 was greater when preceded by sucrose application (Fig. 3e and 85.1 ± 6.1 pA versus 51.6 ± 5.4, *n* = 11 cells from 6 mice, *P* = 0.001, paired *t*-test). This corresponded to an increase in extracellular glutamate of 19 nM (Fig. 3f and 52.5 ± 7.2 nM versus 33.4 ± 3.9 nM; *n* = 10 cells from 6 mice, *P* = 0.009). Both the increase in mEPSC frequency and the increase in D-AP5-sensitive current were small in absolute terms, yet resolvable with our technique. These results demonstrate that NMDAR-mediated current is a sensitive measure of extracellular glutamate, and can report sub-micromolar changes attributable to increased vesicular release. We conclude that if CB1R activation leads to astrocytic glutamate release, it must be either be rare, i.e., occurring in only a small subset of astrocytes, or minimal, i.e., involve very little glutamate, or both.

The question remains, then, of whether CB1R-dependent tLTD requires astrocytic glutamate release. In L2/3, CB1R activation reliably increased astrocytic calcium transients; however, only a small subset of astrocytes contain the necessary machinery to package and release glutamate^28^. Because tLTD is a robust phenomenon, this raises the question of whether the salient signal is something other than astrocyte-derived vesicular glutamate. In several preparations, ATP/adenosine^12,29,30^ and d-serine^31^ have been identified as mediators of the downstream effects of astrocytic CB1R activation, yet in other cases, the soluble messenger(s) remain unknown^32^. Given that astrocyte calcium signals have been linked with an vast array of effects and effectors^32–34^, it remains possible that L2/3 astrocytes regulate tLTD induction through a mechanism other than vesicular glutamate release.

## Methods

### Acute slice preparation

CD1 or C57/BL6 mice of both sexes (postnatal day 12–29) were acquired from Charles River. All methods were carried out in accordance with German animal welfare laws. All experimental procedures were approved by the Institutional Animal Care and Ethics Committees of the University of Göttingen (T22.20). Mice were deeply anesthetized with isoflurane and decapitated. The brain was rapidly removed into ice-cold physiological^35^ artificial cerebrospinal fluid (ACSF) consisting of (in mM): 119 NaCl, 26 NaHCO_3_, 10 glucose, 3 Na-pyruvate, 1.3 Na-ascorbate, 1 NaH_2_PO_4_, 4.2 KCl, 1.2 CaCl_2_, 0.7 MgCl_2_, continuously bubbled with 95%/5% O_2_/CO_2_ (pH 7.39, 290 mOsm). The tissue was then blocked and affixed to the slicing platform of a Leica VT1200S vibratome with cyanoacrylate glue (Loctite). Coronal slices (270 μm) containing barrel cortex were transferred to a heated chamber, where they were kept at 37 °C in ACSF until use, up to 7 h after slicing.

### Electrophysiology

Neurons were identified using gradient-contrast video microscopy. Whole-cell recordings were obtained using Schott 8250 glass patch pipettes (open tip resistance 2–6 MΩ). The internal solution consisted of (in mM): 130 Cs-methanesulfonate, 5 NaCl, 10 HEPES, 4 MgCl_2_, 14 Na-phosphocreatine, 4 Na-ATP, Na-0.4 GTP, 0.1 EGTA; pH adjusted to 7.3 with CsOH, 300 mOsm. In a subset of the experiments shown in Fig. 1 (8/13), 100 µM CdCl_2_ was included in the ACSF to block voltage-sensitive calcium channels (VSCCs). For all other experiments in which cells were voltage-clamped at +30 mV, verapamil (0.2 mM) was included in the internal solution to block VSCCs. Data obtained using the two different methods were pooled, as no difference was detected (estimated glutamate concentration with external CdCl_2_: 26.8 ± 3.8 nM, *n* = 8 cells from 5 mice; with internal verapamil: 30.7 ± 6.4 nM, *n* = 5 cells from 5 mice; *P* = 0.59, unpaired *t*-test).

Miniature excitatory postsynaptic currents (mEPSCs) were recorded at -70 mV in the presence of TTX (0.5 µM) to block action potential generation, D-AP5 (50 µM) to block NMDARs, and picrotoxin (50 µM) to block GABA_A_Rs. Recordings were made in 10-second blocks, each with a -5 mV, 20 ms long voltage step to monitor series resistance. Traces were analyzed offline using a template-matching function^36^. The frequency of mEPSCs was calculated by counting the events during seven consecutive recording blocks. During sucrose application, mEPSC frequency was measured beginning one minute after sucrose solution entered the recording chamber. Wash measurements were made beginning 8.5 min after cessation of drug application.

Electrophysiological recordings were filtered at 2 kHz and digitized at 5 kHz for standing current experiments, or filtered at 2.6–3 kHz and digitized at 10 kHz for mEPSC experiments, using a Multiclamp 700B amplifier (Molecular Devices) controlled with Prairieview 5.4 software (Bruker). Data were analyzed using IgorPro 8 (Wavemetrics). Reported membrane potentials have not been corrected for the junction potential (-8 mV relative to ACSF). All experiments were performed in continuously bubbled ACSF (2 ml/min) at 33–35 °C.

### Solution delivery

For the experiments testing the effect of sucrose on mEPSC frequency (Fig. 3a-c), sucrose-containing ACSF was bath-applied. In all other experiments, drugs were delivered to the slice via flow-pipe to decrease application time and improve washout. The flow-pipe setup consisted of a gravity-fed perfusion system (AutoMate Scientific) connected to a four-way perfusion manifold (MP-4, Warner Instruments). The flow-pipe was constructed from polyimide-coated quartz tubing with an inner diameter of 530 μm (Trajan Scientific and Medical) and was positioned 1 mm upstream of the recorded cell, with the upper edge 600 μm above the surface of the slice. Solutions were delivered at a rate of ~ 0.2 ml/min.

### Pharmacology

AM251, d-(-)-2-Amino-5-phosphonopentanoic acid (D-AP5), 2,3-dioxo-6-nitro-1,2,3,4-tetrahydrobenzo[f]-quinoxaline-7-sulphonamide (NBQX, disodium salt), and tetrodotoxin citrate (TTX), and were purchased from Hello Bio; verapamil hydrochloride and DL-TBOA (TBOA) from Bio-Techne; d-serine from Sigma; picrotoxin from Abcam; WIN 55,212-2 mesylate (WIN) from Biomol; and arachidonyl-2’-chloroethylamide (ACEA) from Tocris. Stock solutions of AM251, picrotoxin, TBOA, and WIN were prepared with DMSO.ACEA was dissolved in ethanol. All other drugs were dissolved in water. All stock solutions were made up at 1000-10000X final concentration and were diluted in ACSF on the day of experiment. Vehicle final concentration (DMSO or ethanol) was 0.0125%.

### Extrapolation of glutamate concentration from NMDAR current

To convert D-AP5-sensitive NMDAR current into an equivalent glutamate concentration, we used the method of Herman and Jahr^19^. First, we measured the current blocked by D-AP5. Next, we measured the response to a standard, sub-saturating concentration of NMDA. This allows estimation, using previously published dose-response relationships^19,20^ (EC_50_ of 37.7 µM, Hill coefficient = 1.3), of the maximal NMDA-evoked NMDAR current for each cell. This current was then scaled up by a factor of 1.77 to account for the higher efficacy of glutamate. The D-AP5-sensitive current relative to the maximal computed glutamate current was then used to estimate glutamate concentration from the glutamate dose response curve (EC_50_ of 1.8 µM, Hill coefficient = 1.3).

### Experimental design and statistical analysis

We confirm this study is reported in accordance with the ARRIVE (Animal Research: Reporting of In Vivo Experiments) guidelines as outlined at https://arriveguidelines.org. Values are presented as mean ± standard error of the mean (s.e.m.). For experiments testing the effect of CB1R agonists ACEA and WIN (Fig. 2, data collection and analysis were performed blind to condition. No statistical methods were used to predetermine sample sizes, but our sample sizes are similar to those generally employed in the field. Differences between conditions were assessed using two-tailed Student’s paired or unpaired *t*-test, and the threshold for significance was set at 0.05. In figure legends, statistical notation is as follows: ns – *p* > 0.05; ** *p* < 0.01; *** *p* < 0.001.

## Data Availability

The datasets used and/or analysed during the current study available from the corresponding author on reasonable request.
